# A comparative study of the metal binding behavior of alanine based bis-thiourea isomers

**DOI:** 10.1186/s13065-017-0304-2

**Published:** 2017-08-04

**Authors:** Imran Fakhar, Bohari M. Yamin, Siti Aishah Hasbullah

**Affiliations:** School of Chemical Sciences and Food Technology, Faculty of Science and Technology, Universiti Kebangsaan Malaysia, 43600 UKM Bangi, Selangor Malaysia

**Keywords:** Bis-thiourea isomers, Binding study, α- and β-alanine, Metal cations

## Abstract

Two new symmetrical bis-thiourea, 2,2′-[{(terephthaloylbis(azanediyl)bis(carbonothioyl) bis(azanediyl)}dipropanoic acid] (1A) and 3,3′-[{(terephthaloylbis(azanediyl)bis (carbonothioyl)bis(azanediyl)} dipropanoic acid] (1B) were synthesized by the reaction of terephthaloyl chloride with α- and β-alanine in good yields. Their binding properties were investigated with various metal cations using UV–Vis titration experiments. Both isomers exhibited effective binding with Ag^+^, Cu^2+^, Hg^2+^, Pb^2+^, Fe^2+^ and Fe^3+^ cations. However, in the presence of other cations, such as Na^+^, Ni^2+^, Co^2+^, Cd^2+^, Zn^2+^, Mn^2+^, Mg^2+^, Ca^2+^, Sn^2+^, Al^3+^, and anions tetrabutylammonium Cl^−^ and H_2_PO_4_^−^, no interaction occurred. Both isomers displayed similar trends towards binding with metal cations.

## Introduction

Thiourea is an analogue of urea and was first synthesized by Nencki [1]. Since then, thiourea compounds have extensively been used as the building blocks of heterocyclic analogues [2]. Amongst this class of compounds, benzoyl derivatives of thiourea have gained a great deal of importance in the present day. Thiourea linkages have contributed greatly to the observed enhancement in various activities [3], including antiviral [4], antibacterial [5, 6], antifungal [7], antitubercular [8, 9], herbicidal [10], insecticidal [11], pharmacological properties [12], as chelating agents [13, 14] and as anticancer compounds [15]. In addition, benzoyl thiourea derivatives have often been used in analytical and biological applications [16, 17].

Amino acids and their derivatives are significant constituents of chemical entities found within many natural frameworks. The synthesis of biologically active amino acid-coupled derivatives has recently become of major interest [18–22].

Thiourea and their amino acid derivatives coordinate to several transition metal ions to form stable complexes. Early useful suggestions of metal ions binding was provided by the old discipline of metal coordination chemistry by Werner [23]. Thioureas, along with its derivatives, are versatile ligands, able to coordinate to metal centers as neutral ligands, monoanions, or dianions [24, 25]. According to Pearson’s hard and soft acid–base concept thiourea, being a soft base, shows an affinity to bind with soft acids like mercury, copper, silver, cadmium ions. Conversely, amino acids, having carboxylic acid functionality, prefer interactions with hard acids like iron, lead, aluminum ions [26]. The thiourea-based derivatives have the ability to coordinate with several metal ions but have not been much explored as receptors for the detection of transition metal ions, this despite both urea and thiourea derivatives being frequently used as anion binding receptors owing to their ability to act as hydrogen-bond donors [27, 28]. However, recently some thiourea-based derivatives and thiourea-based nanoparticles have been used to detect metal ions [29, 30]. In view of these observations, the synthesis of two bis-thiourea isomers having alanine linkers were planned followed by a comparative study of their binding interactions against sixteen metal cations (four soft, six mild and six hard ions) and two tetrabutyl ammonium anions. Both isomers were characterized by IR spectroscopy, ^1^H and ^13^C NMR spectroscopy, ESI–MS, and elemental analysis. Isomer 1B was further confirmed by X-ray crystallography. Binding studies of both isomers were studied by conducting titration experiments using UV–Vis spectroscopy.

## Experimental

### Materials and measurements

All the chemicals were obtained from ACROS Organics (Geel, Belgium) and Sigma-Aldrich (Saint Louis, MO, USA), and were utilized without further purification. All solvents were distilled from CaH_2_ before use. Open tube capillary method was used to determine the melting points utilizing an Electrothermal 9100 (Electrothermal, Southend, England) and were uncorrected. The micro elemental investigation for CHNS were performed using a Carlo Erba 1108 Elemental Analyzer (Milan, Italy). The IR spectra of the isomers were obtained by KBr disc method and were recorded on a Perkin Elmer Spectrum GX spectrophotometer (Perkin Elmer, Waltham, MA, USA) in the range of 400–4000 cm^−1^ with resolution 4 cm^−1^. UV–Vis estimations were performed on double beam Varian UV 3.0 (Cary 100, Varian Australia Pty. Ltd.) spectrophotometer with a quartz cell (1 cm path length) in the scope of 200–800 nm with the highest resolution of 1 nm. Nuclear Magnetic Resonance experiments (^1^H and ^13^C NMR spectra) were done on a Bruker 400 MHz instrument using DMSO-d_6_ as solvent. ESI–MS spectra were recorded on a Micro Tof Q (Bruker, AXS Incorporation, and Madison, WI, USA). Single crystal X-ray experiments were performed on a Bruker D-QUEST diffractometer (Bruker, AXS Inc., Madison, WI, USA) using graphite-monochromated Mo-Kα radiation (λ = 0.71073 Å). Intensity data were measured at room temperature by the ω-scan. Accurate cell parameters and orientation matrix were determined by the full-matrix least-squares fit of 25 reflections. Intensity data were collected for Lorentz and polarization effects. Empirical absorption correction was carried out using multi-scan. The structure was solved by direct methods and least-squares refinement of the structure was performed by the SHELXL-2007 program [31]. All the non-hydrogen atoms were refined anisotropically. The hydrogen atoms were set in the calculated positions aside from the terminal N-atoms of thiourea moiety located from Fourier maps and refined isotropically [32].

### General procedure for the synthesis of isomers (1A and 1B)

**Scheme 1:**
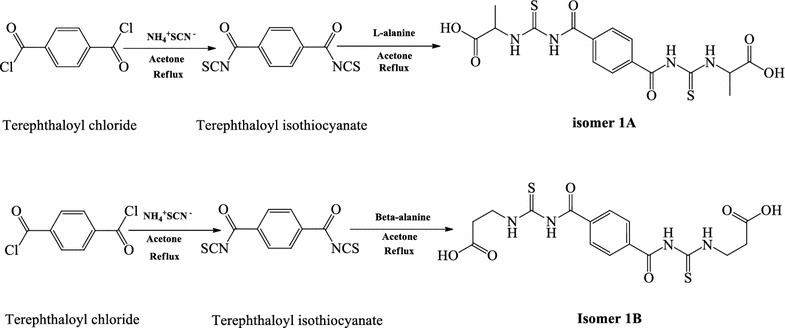
Synthesis of bis-thiourea alanine based isomers 1A and 1B

Benzene-1,4-dicarbonyl chloride (terephthaloyl chloride) (0.609 g, 0.003 mol), was dissolved in dry acetone (20 ml). A solution of ammonium thiocyanate (0.456 g, 0.006 mol), antecedently dried (80 °C, 2 h) in dry acetone (15 ml) was prepared. Ammonium thiocyanate was added slowly to the stirring solution of benzene-1, 4-dicarbonyl chloride, and the reaction mixture was stirred at room temperature for 1 h. The white precipitate of ammonium chloride were filtered off. α- or β-alanine (0.534 g, 0.006 mol) in dry acetone (15 ml) was added to the filtrate containing benzene-1,4-dicarbonyl isothiocyanate intermediate. The reaction mixture was then refluxed for 24–30 h. The solution was allowed to cool to RT and an excess of crushed ice added to the flask, bis-thiourea analogues 1A and 1B were collected as precipitates which were then washed several times with water and dried in a desiccator (using calcium sulfate as a drying agent). Both analogues were recrystallized from ethanol/DMSO to afford 1A and 1B in good yield (89.1 and 91.8%, respectively, Scheme 1).

## Results and discussion

### Characterization

#### 2,2′-[{(terephthaloylbis(azanediyl)bis(carbonothioyl)bis(azanediyl)} dipropanoic acid] (**1A**)

Using the general method outlined above, compound 1A was isolated as a yellowish solid (0.760 g, 89.1%), mp: 214–215 °C, [Found: C, 44.99; H, 4.19; N, 13.11; S, 15.01; O, 22.7%; M^+^, 449.07. C_16_H_18_N_4_O_6_S_2_ requires C, 45.06; H, 4.25; N, 13.14; S, 15.04; O, 22.51%]; *ν*_max_ (KBr/cm^−1^) 3358 (N–H), 3180 (C–H_arom_), 2929 (C–H_aliph_), 1728 (C=O), 1676 (COOH), 1545 (C–N), 1521 (Ar–C), 1012 (C=S); δH (400 MHz, DMSO-d_6_, 1.50 (6H, d, J = 7.2 Hz, 2×CH_3_), 4.83 (2H, quint, J = 7.2 Hz, 2×CH), 8.00 (4H, s, Ar–H), 11.24 (2H, d, J = 6.8 Hz, 2×NH), 11.74 (2H, s, 2×NH). δC (100 MHz, DMSO-d_6_) 17.5 (CH_3_), 53.5 (CH), 129.0 (CH_arom_), 136.3 (C_arom_), 168.2 (C=O), 173.3 (COOH), 180.1 (C=S); MS (EI): (*m*/*z*) = 449.07 [M + Na]^+^.

#### 3,3′-[{(terephthaloylbis(azanediyl)bis(carbonothioyl)bis(azanediyl)} dipropanoic acid] (**1B**)

Using the general method outlined above, compound 1B was isolated as a white solid (0.784 g, 91.8%) as a white solid, mp: 203–204 °C, [Found: C, 45.09; H, 4.31; N, 13.01; S, 15.03; O, 22.56%; M^+^, 449.47. C_16_H_18_N_4_O_6_S_2_ requires C, 45.06; H, 4.25; N, 13.14; S, 15.04; O, 22.51%]; ν_max_ (KBr/cm^−1^) 3330 (N–H), 3245 (C–H_arom_), 2950 (C–H_aliph_), 1711 (C=O), 1670 (COOH), 1554 (C–N), 1527 (Ar–C), 1025 (C=S); δH (400 MHz, DMSO-d_6_, 2.65 (4H, t, J = 6.0 Hz, 2×CH_2_), 3.82 (4H, d, J = 6.0 Hz, 2×CH_2_), 7.95 (4H, s, Ar–H), 10.99 (2H, t, J = 5.6 Hz, 2×NH), 11.49 (2H, s, 2×NH). δC (100 MHz, DMSO-d_6_) 32.6 (CH_2_), 41.0 (CH_2_), 127.8 (CH_arom_), 129.0 (C_arom_), 168.0 (C=O), 173.4 (C=OOH), 180.5 (C=S); MS (EI): (*m*/*z*) = 449.47 [M + Na]^+^.

### IR spectroscopy

IR spectra of both isomers were in accordance with the vibrational frequencies of the functional groups as found in the literature [3, 46]. The N–H stretching vibrations were observed in the range 3330–3358 cm^−1^. The O–H stretching frequencies of the carboxylic groups were overlapped by N–H stretching peak and hence could not be observed. The C–H stretching vibrations for the sp^2^ carbon of the aromatic ring of both isomers were observed in the range 3180–3245 cm^−1^ [33] whereas, the C–H stretching vibrations for the sp^3^ mode of the alkyl chain were observed in the range 2930–2950 cm^−1^ [34]. The frequency for the C=O and C=O_carboxylic_ stretches were observed at 1728, 1676, 1711, and 1670 cm^−1^ for the isomers 1A and 1B, respectively [35]. The *ν* (C–N) and *ν* (C=C_aromatic_) vibrational frequencies were observed at 1545, 1521 and 1554, 1527 cm^−1^ for isomers 1A and 1B, respectively. All of the values mentioned were found in accordance with those reported [3]. The *ν* (C=S) vibrational frequencies for both isomers were observed at 1012 and 1025 cm^−1^. The lowering in the vibrational frequencies of (C=S) bonds were due to mesomeric electron releasing effect of the nitrogen bonded to the thiocarbonyl group (N–C=S). This lowering of C=S stretching frequencies is due to an acquiring of a partial polar character [36].

### ^1^H NMR and ^13^C NMR spectroscopy

Bis-thiourea isomers were further characterized and confirmed by ^1^H, and ^13^C NMR. The proton chemical shifts of the amide functionality appeared as a singlet at δ 11.74 and 11.49 ppm for isomers 1A and 1B, respectively. The thioamide protons were observed as doublets at δ 11.24, 10.99 ppm for the isomers 1A and 1B, respectively. The downfield signals of both amide and thioamide protons are due to the formation of H-bonding between the amino proton and the oxygen/sulfur atoms of carbonyl/thiocarbonyl group, as well as the anisotropic effect [37]. All the aromatic protons for both isomers were identical and found as singlets at δ 8.0 and 7.95 ppm for 1A and 1B, respectively. The chemical shift for the proton on the chiral carbon of isomer 1A was observed at δ 4.83 ppm. The signal was observed downfield due to the deshielding effect of the nearby electron withdrawing thioamide group as well as the anisotropic effect of the carboxylic carbonyl group. Isomer 1B contains no source of chirality and so two methylene groups are present. The methylene group proximal to the carboxylic acid were observed downfield at δ 3.82 ppm as a doublet due to the anisotropic effect of the carbonyl group. Protons of the second methylene group were observed as a triplet at δ 2.65 ppm slightly downfield due to deshielding from the electron withdrawing thioamide group. The methyl protons for isomer 1A were observed as a doublet at δ 1.53 ppm.

The ^13^C NMR spectra for both isomers 1A and 1B were in accordance with those that have been reported previously [38]. The carbon chemical shifts of C=S, C=C_arboxylic_ and C=O were found at δ 180.1, 173.3 and 168.2 ppm for isomer 1A and at δ 180.5, 173.4 and 168.0 for isomer 1B, respectively. The aromatic carbons were observed at δ129.0 and 136.3 ppm for isomer 1A and at δ 127.8 and 129.0 ppm for isomer 1B, respectively. The signal for the chiral carbon of isomer 1A was observed at δ 53.5 ppm and that of the carbon bearing the methyl group at δ 17.5 ppm. Whereas the chemical shifts of two methylene groups of isomer 1B were observed at δ 3.82 and 2.65 ppm, respectively.

### Elemental analysis and ESI-Mass spectroscopy

The CHNS analysis for both isomers were found to be in close accordance with the theoretical values.

The ESI–MS spectra, for both isomers 1A and 1B, showed sodium molecular ion peaks at *m*/*z* 449, which is in accordance with the expected molecular ion peak values.

### X-ray crystallography of isomer 1B

The isomer 1B crystallized in monoclinic system with space group C2/c, a = 26.9433(13), b = 4.7668(2), c = 15.1750(7), α = 90, β = 100.926(2), γ = 90, Z = 4 and V = 1913.65(15). Crystallographic data for the structure determination has been deposited with the Cambridge Crystallographic Data number CCDC 1518921. The given crystal state and refinement parameters are given in Table 1.

**Table 1:** Crystal data and structure refinement for isomer 1B

Identification code	boly370_0 m
Empirical formula	C_16_H_18_N_4_O_6_S_2_
Formula weight	426.46
Temperature	303(2) K
Wavelength	0.71073 Å
Crystal system	Monoclinic
Space group	C2/c
Unit cell dimensions	a = 26.9433(13) Å; α = 90°
	b = 4.7668(2) Å; β = 100.926(2)°
	c = 15.1750(7) Å; γ = 90°
Volume	1913.65(15) Å3
Z	4
Density (calculated)	1.480 Mg m^−3^
Absorption coefficient	0.320 mm^−1^
F(000)	888
Crystal size	0.49 × 0.36 × 0.11 mm^3^
Theta range for data collection	2.87–28.31°
Index ranges	−35 <= h <= 35, −6 <= k <= 6, −18 <= l <= 20
Reflections collected	29,791
Independent reflections	2376 [R(int) = 0.0372]
Completeness to theta = 28.31°	99.7%
Absorption correction	Semi-empirical from equivalents
Max. and min. transmission	0.9656 and 0.8588
Refinement method	Full-matrix least-squares on F^2^
Data/restraints/parameters	2376/0/128
Goodness-of-fit on F^2^	1.064
Final R indices [I >2 sigma(I)]	R1 = 0.0538, wR2 = 0.1507
R indices (all data)	R1 = 0.0673, wR2 = 0.1618
Largest diff. peak and hole	0.335 and −0.357 e Å^−3^

**Fig. 1:**
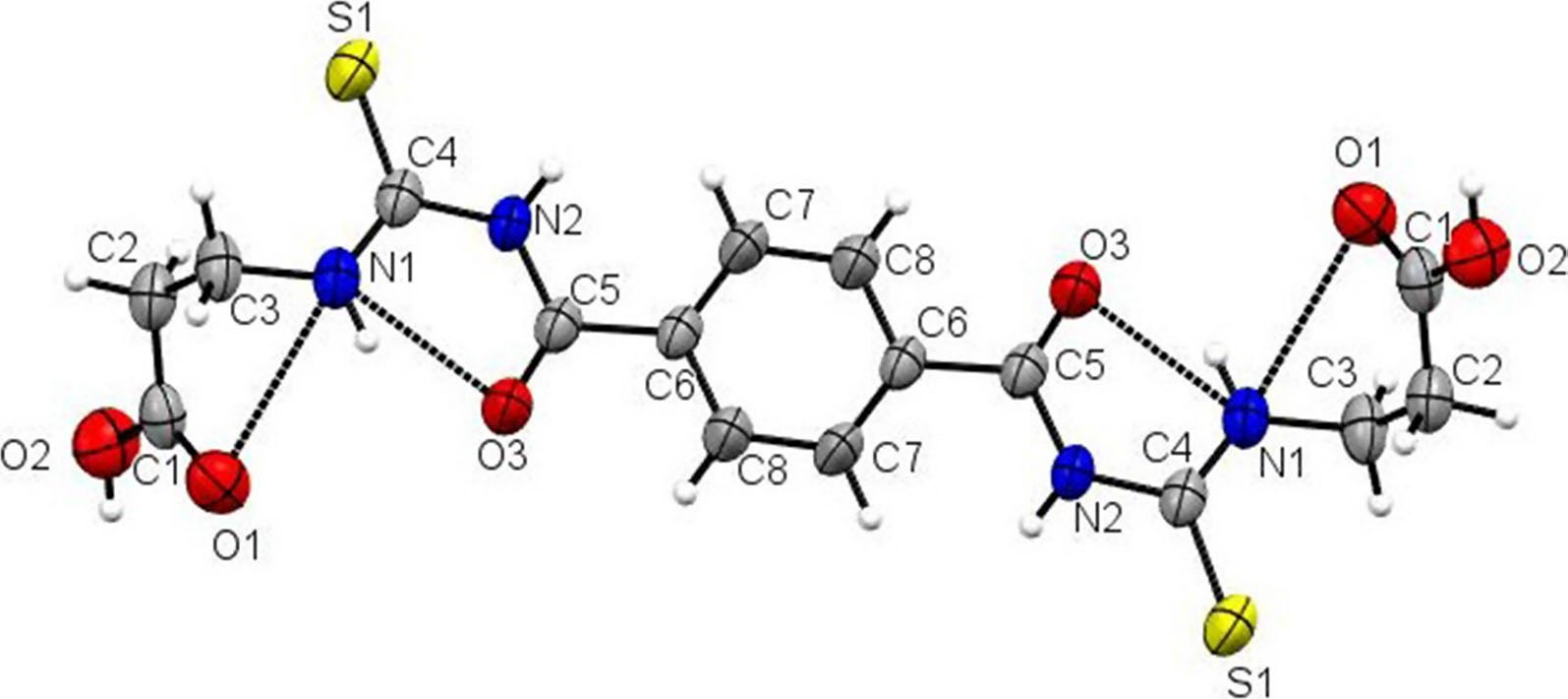
ORTEP diagram of the 3, 3′-[{(terephthaloylbis(azanediyl)bis(carbonothioyl)bis (azanediyl)}dipropanoicacid]. **1B** was drawn at 50% probability displacement ellipsoids. The *dashed line* indicates the intramolecular hydrogen bond

The molecule 1B adopts a cis–trans configuration with respect to the position of the propionic acid relative to the S_1_ atom across the C(4)–N(1) bonds. Figure 1 shows the conformational structure of the molecule with atoms numbered.

The thiourea fragment, S(1)/N(1)/N(2)/O(3)/C(5) and benzene ring are planar with maximum deviation of 0.073(2) Å for the N(1) atom from the least-squares plane of the thiourea fragment. The thiourea moiety along with benzene ring makes an angle of 90.0(3)° with the propionic acid fragment (Table 2). The bond lengths and angles in isomer 1B is within normal ranges [39, 40].

**Table 2:** Selected bond lengths (Å) and bond angles (°) for isomer 1B

Bond	Length (Å)	Bond	Angles (°)
S(1)–C(4)	1.672(2)	C(4)–N(1)–C(3)	123.7(2)
O(1)–C(1)	1.212(3)	C(5)–N(2)–C(4)	126.52(18)
O(2)–C(1)	1.313(3)	O(1)–C(1)–O(2)	122.1(2)
O(3)–C(5)	1.214(3)	O(1)–C(1)–C(2)	124.6(2)
N(1)–C(4)	1.316(3)	O(2)–C(1)–C(2)	113.26(19)
N(1)–C(3)	1.464(3)	C(1)–C(2)–C(3)	112.6(2)
N(2)–C(5)	1.377(3)	N(1)–C(3)–C(2)	111.31(19)
N(2)–C(4)	1.399(2)	N(1)–C(4)–N(2)	116.77(18)
C(1)–C(2)	1.494(4)	N(1)–C(4)–S(1)	122.86(16)
C(2)–C(3)	1.514(3)	N(2)–C(4)–S(1)	120.35(16)
C(5)–C(6)	1.500(3)	O(3)–C(5)–N(2)	122.32(18)
C(6)–C(7)	1.371(3)	O(3)–C(5)–C(6)	120.38(19)
C(6)–C(8)	1.377(3)	N(2)–C(5)–C(6)	117.30(19)
C(7)–C(8)#1	1.382(3)	C(7)–C(6)–C(8)	118.52(19)
C(8)–C(7)#1	1.382(3)	C(7)–C(6)–C(5)	124.88(18)
		C(8)–C(6)–C(5)	116.59(19)

Symmetry transformations used to generate equivalent atoms: #1 −x,−y,−z

**Fig. 2:**
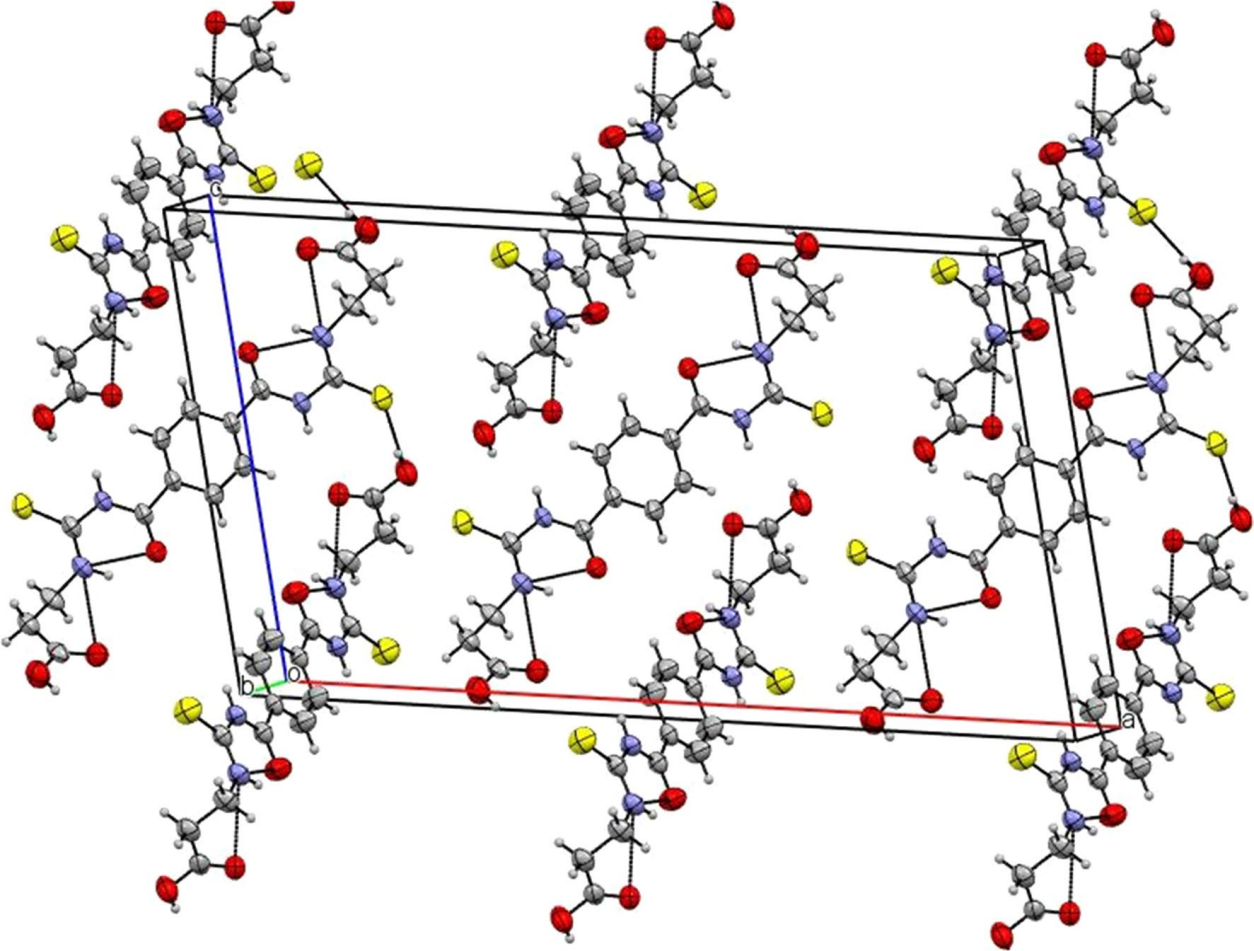
Molecular packing of 1B viewed down the b axis. *Dashed lines* denote C–H….O, O–H….S and N–H….O hydrogen bonds

In the molecule there are three intramolecular H-bonds, N(1)…H(1)…O(3), C(3)…H(3B)…S(1) and C(8)…H(8)…O(3) (Table 3). In the crystal structure, the molecules are linked by O(2)…H(2)…S(1), N(1)…H(2C)…O(1) and C(7)…H(7)…O(1) intermolecular H-bonds forming a 3-D network (Fig. 2).

**Table 3:** Hydrogen bonds for isomer 1B [(Å) and (°)]

D–H…A	d(D–H)	d(H…A)	d(D…A)	<(DHA)
N(2)–H(2C)…O(1)	0.86	2.28	3.126(2)	168
N(1)–H(1)…O(3)	0.86	1.92	2.603(2)	134.9
O(2)–H(2)…S(1)	0.82	2.26	3.072(2)	174
C(3)–H(3B)…S(1)	0.97	2.64	3.042(3)	105
C(7)–H(7)…O(1)	0.93	2.20	3.123(3)	174
C(8)–H(8)…O(3)	0.93	2.41	2.737(3)	100

Symmetry transformations used to generate equivalent atoms: #1 −x, −y, −z # 2 x, −y + 1, z − 1/2 #3 x, −y + 1, z+

## Binding studies

### UV–Vis spectra measurements

Firstly, stock solutions for both isomers (1A and 1B) were prepared in DMSO (1 × 10^−3^ M) before making stock solutions for both metal cations and tetrabutylammonium anions, also in DMSO (1 × 10^−3^ M). By adding different volumes (0–600 µl) of metal ions and terabutylammonium anions to a series volumetric flasks, together with an equal volume (100 µl) of the isomers 1A and 1B, the work solutions were prepared. Each of the work solutions were then diluted by adding DMSO and shaken for several minutes. Readings were recorded on UV–Vis spectrophotometer using quartz cuvettes (1 cm path length) in the range of 200–800 nm with the utmost resolution of 1 nm. The correlation coefficient was computed using Pearson product-moment correlation strategy. By plotting a fit line curve using Sigma Plot 12.0 (Systat Software Inc.), dissociation constant (K_d_) values were intended using a nonlinear regression equation. Detection limit was figured by 3 σ/S, where ‘σ’ is the std. deviation and ‘S’ is the incline in the titration curve. To demonstrate the veracity of information, more than 20 arrangements of continuous data were gathered in the UV–Vis titration tests until absorbance values approached equilibrium.

### Theory and calculations

The correlation coefficient was utilized to quantify a linear association between the two factors (absorbance vs concentration) amid the titration tests. The Pearson product-moment correlation strategy was utilized as part of this study to quantify the degree of linear dependence between the two variables. The formula for correlation coefficient ‘r’ can be accomplished by substituting assessments of the covariance and variance in the equation below [47].

$$r = rxy = \frac{{n\left( {\sum {xy} } \right) - \left( {\sum x } \right)\left( {\sum y } \right)}}{{\left( {n\sqrt {\sum {x^{2} - \left( x \right)^{2} } } } \right)\left( {n\sqrt {\sum {y^{2} - \left( {\sum y } \right)^{2} } } } \right)}}$$

where: r = correlation coefficient; x = concentration; y = absorbance; n = no. of observations.

The detection limit was calculated by utilizing the formula.

$$DL = {{3\sigma } \mathord{\left/ {\vphantom {{3\sigma } S}} \right. \kern-0pt} S}$$

where: σ = std. deviation of 5 blank values; S = slope of the fit-line titration curve.

#### Clark’s theory of binding

Alfred Joseph Clark developed this concept in 1926, and mathematically stated that for a bimolecular reaction [48]:

$$H + G\,{\mathbf{ \leftrightarrows }}\,H - G$$

The equilibrium dissociation constant (K_d_) or an equilibrium association constant (K_a_), which are proportionally related, is demonstrated by the following:


Regardless of the mechanism, every reversible reaction achieves equilibrium conveyance of reactants and products when the rates of both the forward and reverse reactions reach equivalence. The general rate can be communicated as:

$$\frac{d[H - G]}{dt} = k_{assn} [H][G] - k_{diss} [H - G].$$

At the beginning of a reaction, the association rate (k_assn_ [H] [G]) would overwhelm. As more of the complex is formed, the association rate would diminish and the dissociation rate would increase. Eventually, the rates of the opposing reactions would become equivalent, and be described as:

$$\frac{d[H - G]}{dt} = \frac{ - d[H]}{dt} = \frac{ - d[G]}{dt} = k_{assn} [H][G] - k_{diss} [H - G] = 0.$$

Under these conditions:

$$\frac{[H][G]}{[H - G]} = \frac{{k_{diss} }}{{k_{assn} }} = K{}_{d}.$$

This expression demonstrates that the equilibrium concentration of reactants and products will have a constant ratio (K_d_) that is equivalent to the proportion of the forward and reverse rate constants. K_d_ is called the equilibrium dissociation constant.

In the present study the dissociation constant (also termed as binding constant (K_d_) was computed by the Nonlinear Regression formula utilizing Sigma plot 12.0 (Systat Software Inc.).

**Fig. 3:**
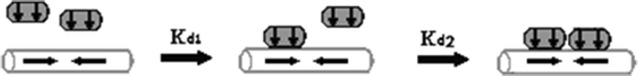
Graphical representation of two-site binding

For the two site mode of binding (Fig. 3), the nonlinear regression equation is expressed as the following:

$$y = B_{\text{max} 1} \cdot \frac{x}{{K_{d1} + x}} + B_{\text{max} 2} \cdot \frac{x}{{K_{d2} + x}}$$

where: B_max_ = host–guest complex; y = absorbance; x = [G]/[H].

### Selectivity of bis-thiourea isomers against cations

**Fig. 4:**
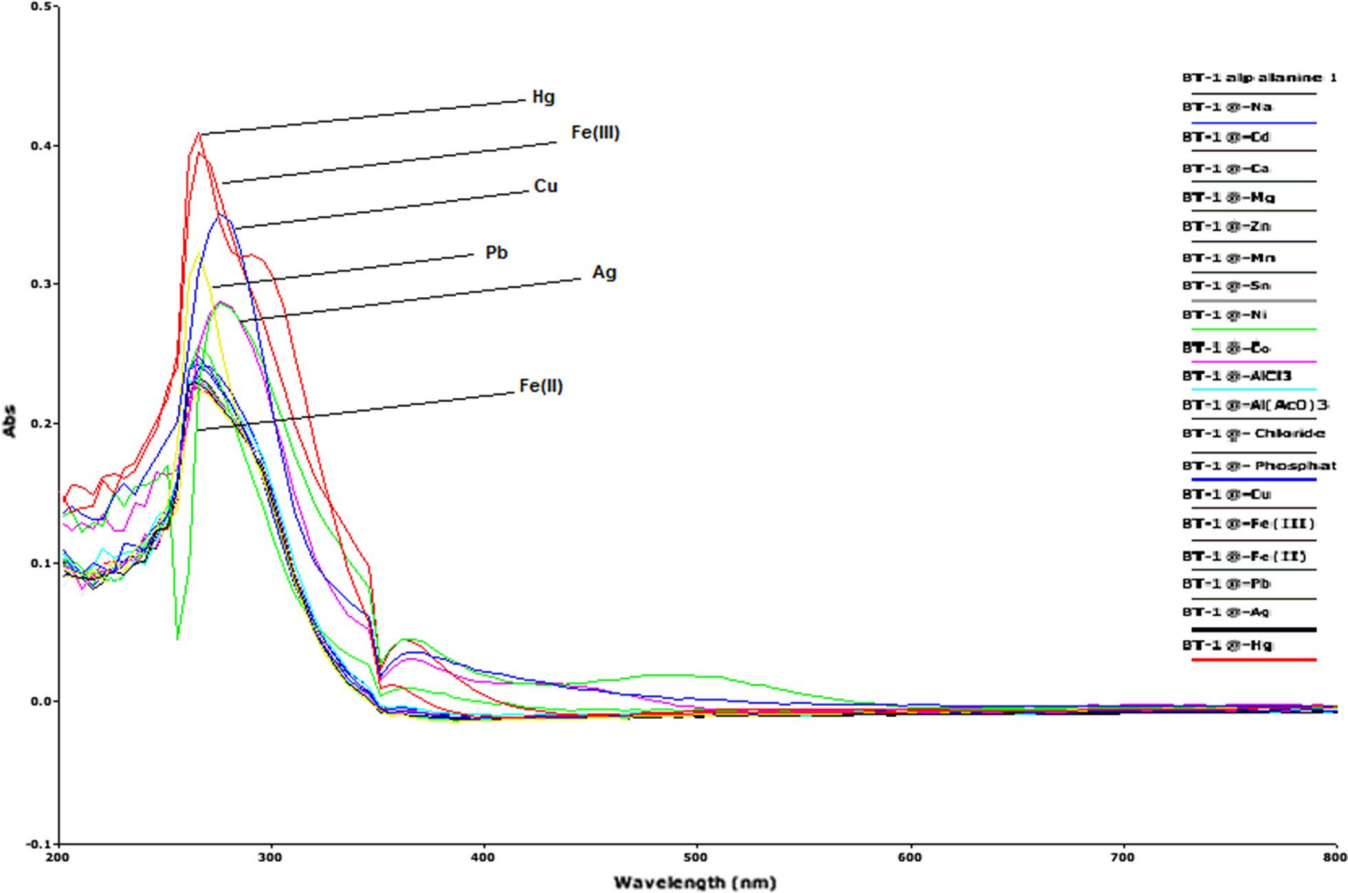
Interactions of isomer 1A with various metal ions and tetrabutylammonium ions

**Fig. 5:**
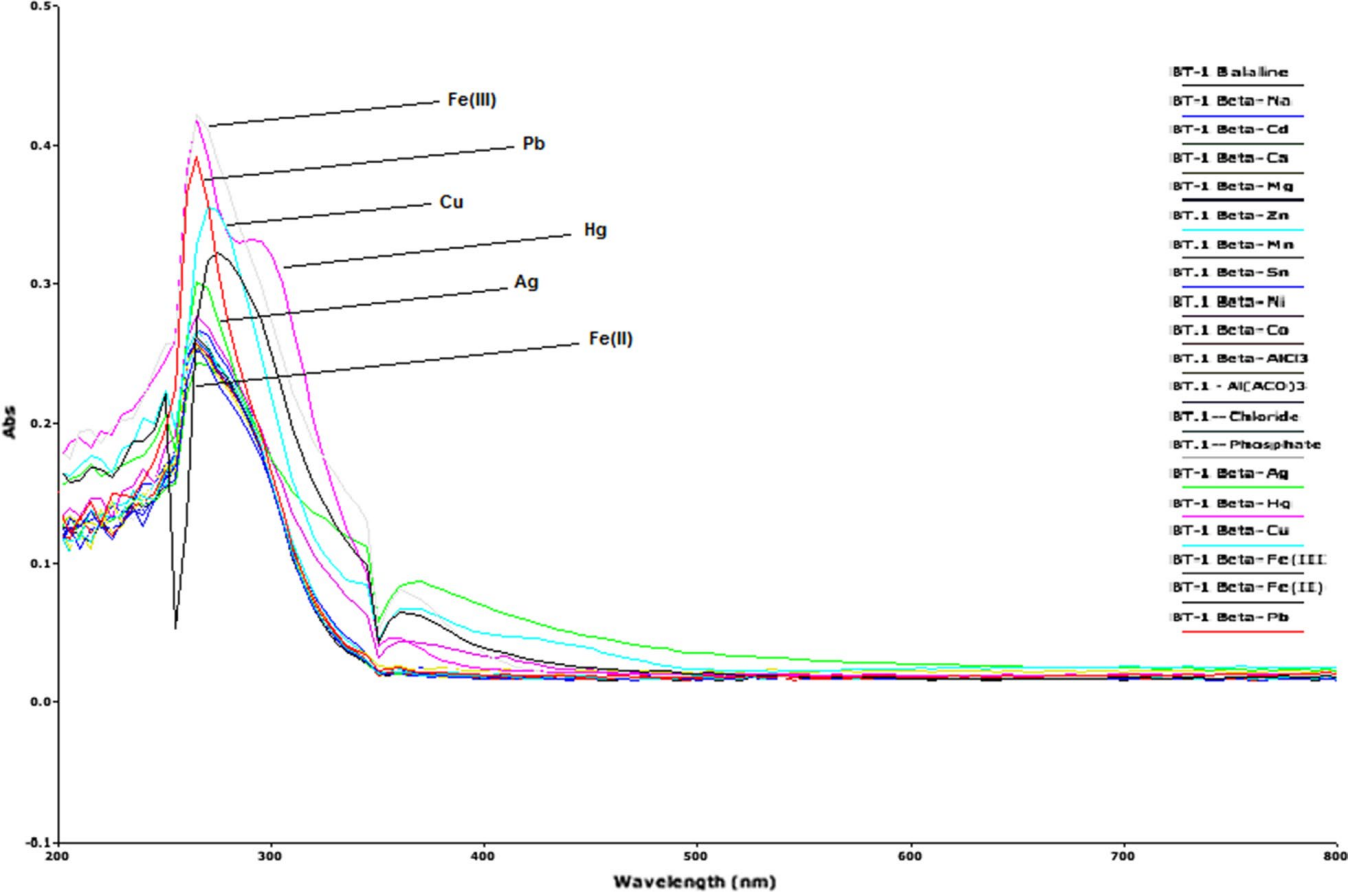
Interactions of isomer 1B with various metal ions and tetrabutylammonium ions

In the first place, the interaction properties of the isomers in DMSO were examined against sixteen metal cations, four of which are soft metal ions such as Ag^+^, Cu^2+^, Co^2+^ and Hg^2+^, six are mild metal ions such as Fe^2+^, Ni^2+^, Pb^2+^, Mn^2+^ and Zn^2+^ and six are hard metal ions such as Na^+^, Ca^2+^, Mg^2+^, Fe^3+^, Cd^2+^, Sn^2+^ and Al^3+^ according to the Pearson scale. The two tetrabutylammonium anions of Cl^−^ and H_2_PO_4_^−^ were also investigated. Both isomers (1A and 1B) did not show any appreciable interactions with both Cl^−^ and H_2_PO_4_^−^ ions. Whereas both isomers showed reasonable interactions with six metal ions, five which are soft to mild (Ag^+^, Cu^2+^, Hg^2+^, Fe^2+^, Pb^2+^) and one which is hard (Fe^3+^). The results of interactions are shown in (Figs. 4, 5) for isomer 1A and 1B, respectively. On the Pearson scale thioureas are considered soft bases and so would be expected to have the most intense interactions with mild to soft Pearson acidic ions.

### Binding behavior and binding mechanism of bis-thiourea isomers

#### Comparison of binding behavior

**Fig. 6:**
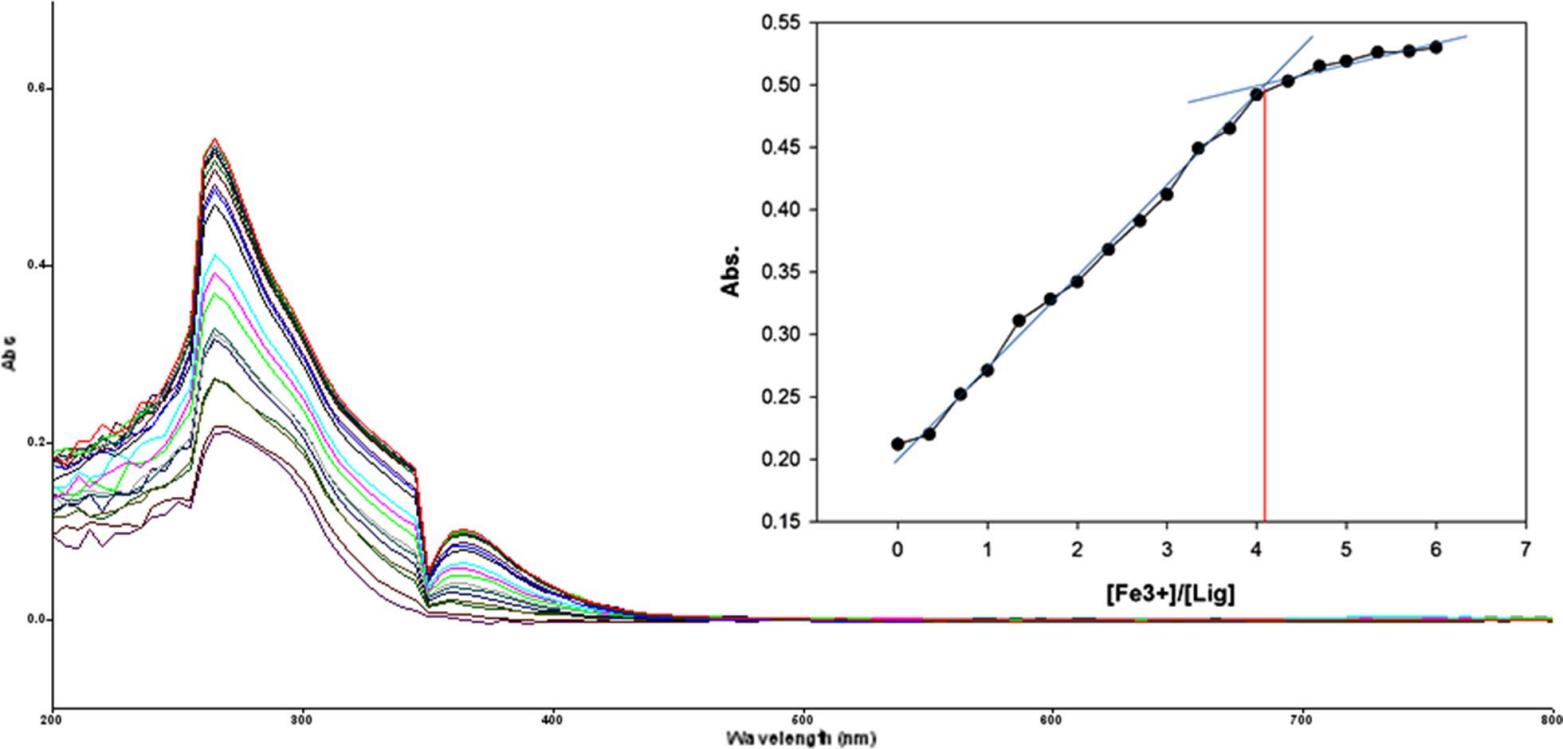
Titration of isomer **1A** vs **Fe** ^**3+**^ (*Inset* Binding behavior + stoichiometry)

**Fig. 7:**
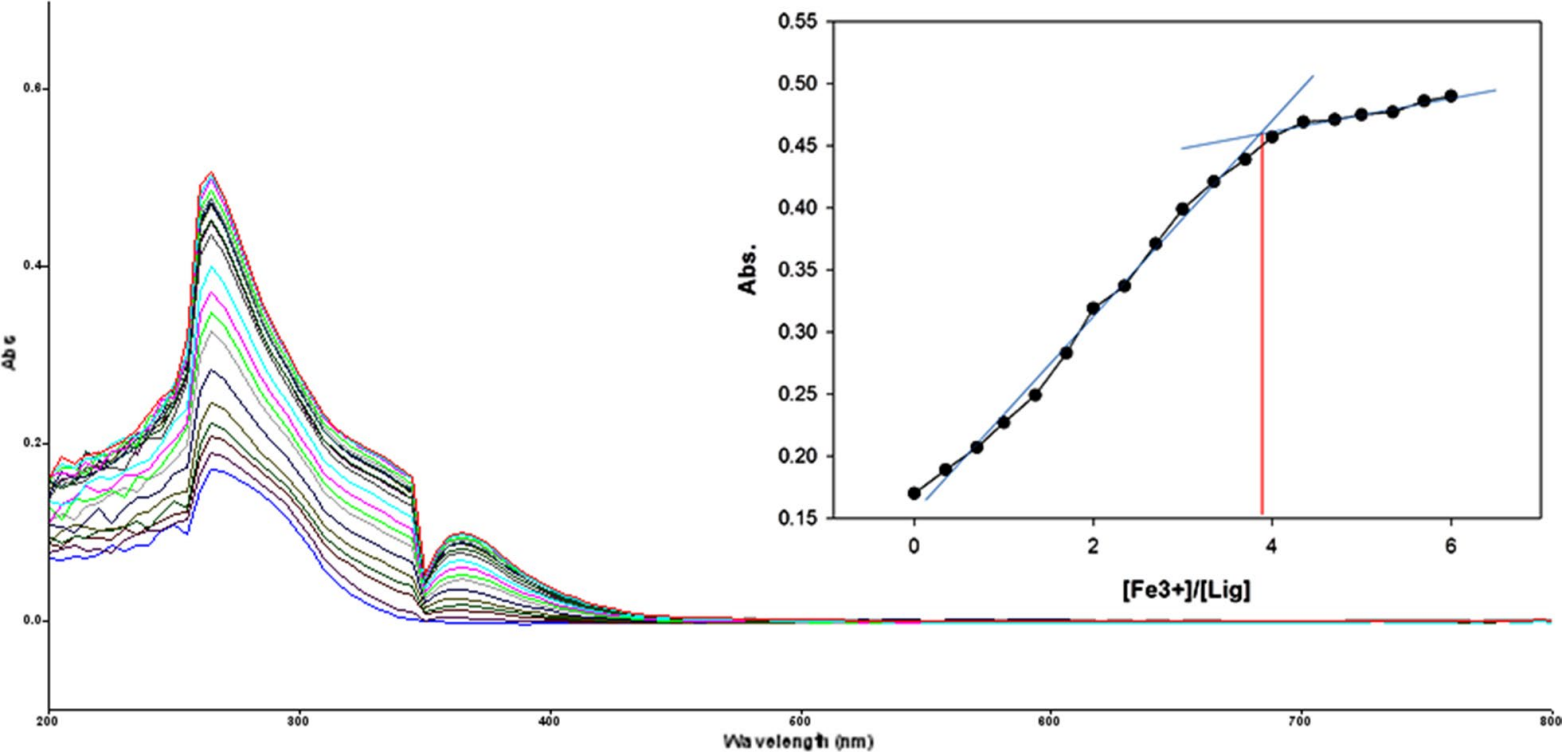
Titration of isomer **1B** vs **Fe** ^**3+**^ (*Inset* Binding behavior + stoichiometry)

To inspect the coupling behavior of isomer 1A and 1B against selected metal cations, titration experiments were carried out. In the control experiment (isomers without metal cations), the absorption maxima of both isomers were seen at 265 nm, which can be allocated to an intramolecular charge transfer (ICT) absorption band as is the known case with thioureas [41]. Upon sequential addition of cations to the test solutions, just Fe^3+^, Fe^2+^, Cu^2+^, Pb^2+^, Hg^2+^, and Ag^+^ gave exceptional enhancement of emission intensity at 265 nm for both isomers 1A and 1B. The increase of emission absorbance intensity was credited to the conceivable formation of host–guest complexes at two probable sites. The first and most likely site of complexation is the carboxylate functionality of α/β-alanine [42], as shown by dissociation constant K_d1_ in Table 1. The second interaction would be from the thiourea functionality via C=S and N–H [43] as shown by dissociation constant K_d2_ (Table 1). By looking at the titration spectra of isomers 1A and 1B vs Fe^3+^, Ag^+^, and Cu^2+^ (Figs. 6, 7, 10, 11, 16, 17), another band can be seen to appear at 360–365 nm, which progressively expanded on incremental addition of metal cations. This is due to the deprotonation of the amino proton by counter anions. Fabrizzi et al. additionally reported a similar outcome for a urea based receptor [44]. The absorbance maxima increased linearly with the concentration of all the chosen cations in a given range (0–600 µl). Table 1 also shows the correlation coefficient values and detection limit values in the light of titration investigations. Titration experiment curves and binding behaviors of isomers 1A and 1B against metal ions are also shown (Fig. 6 through to Fig. 17).

#### Binding mechanism

**Fig. 8:**
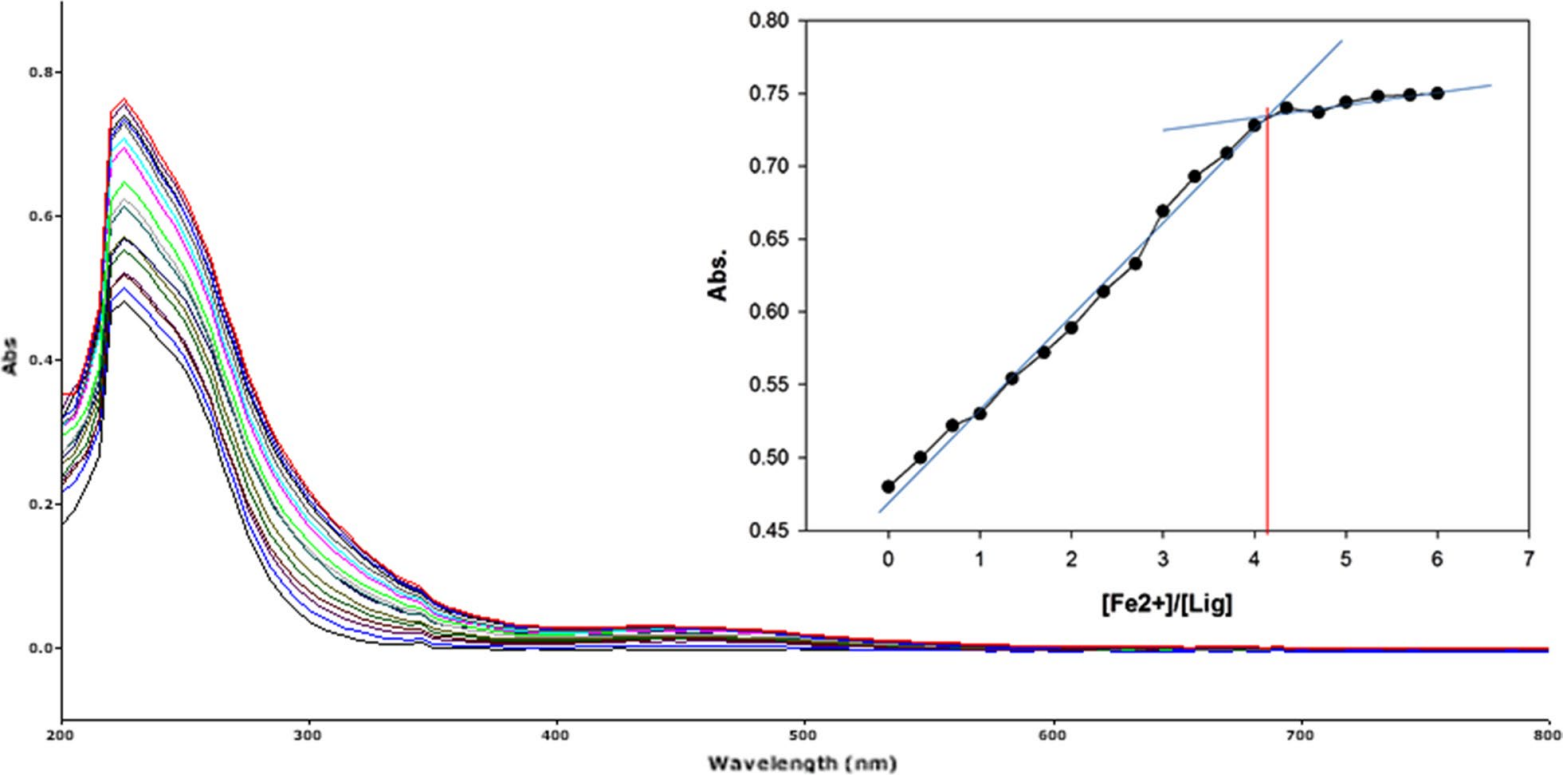
Titration of isomer **1A** vs **Fe** ^**2+**^ (*Inset* Binding behavior + stoichiometry)

**Fig. 9:**
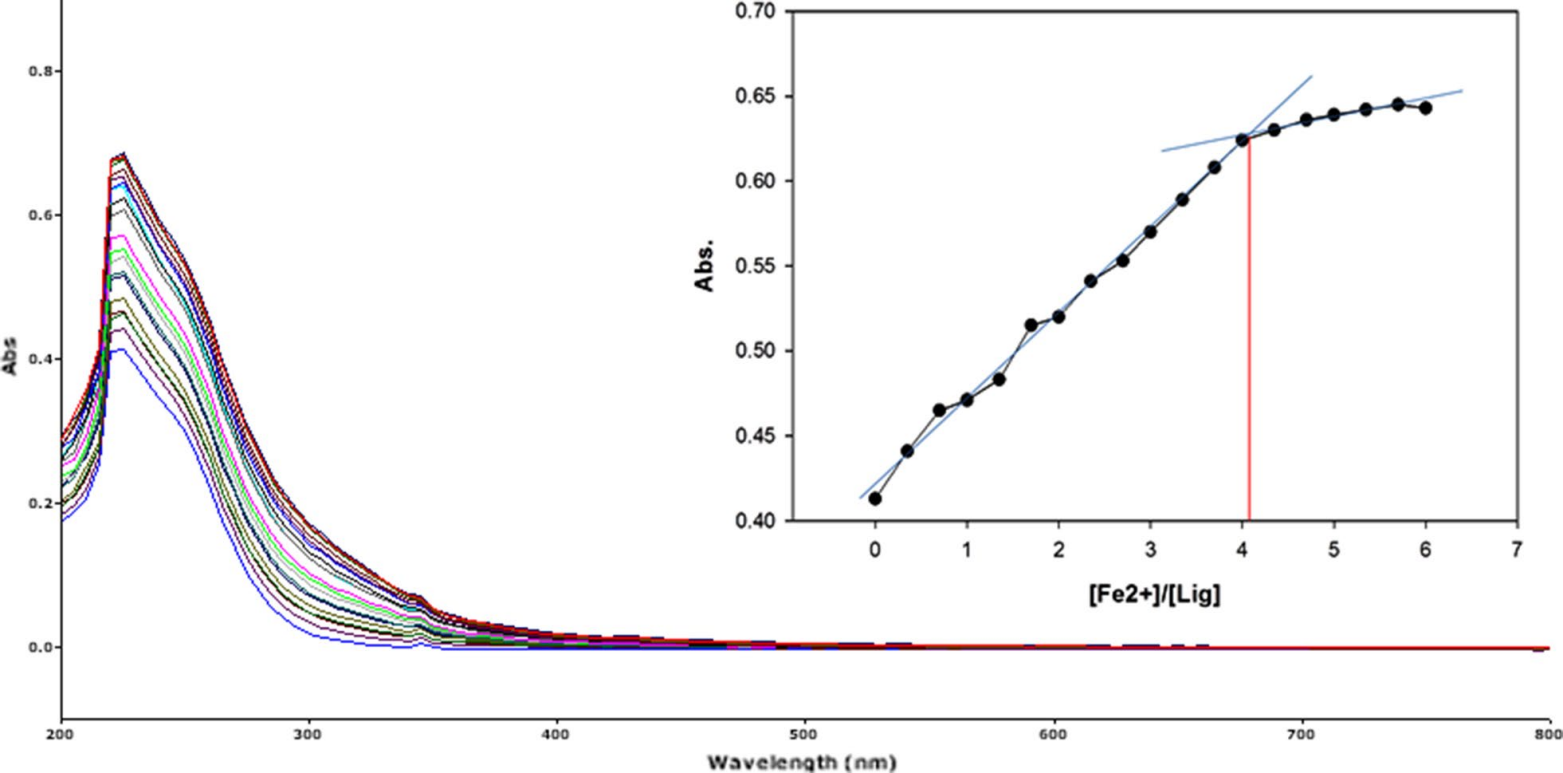
Titration of isomer **1B** vs **Fe** ^**2+**^ (*Inset* Binding behavior + stoichiometry)

**Fig. 10:**
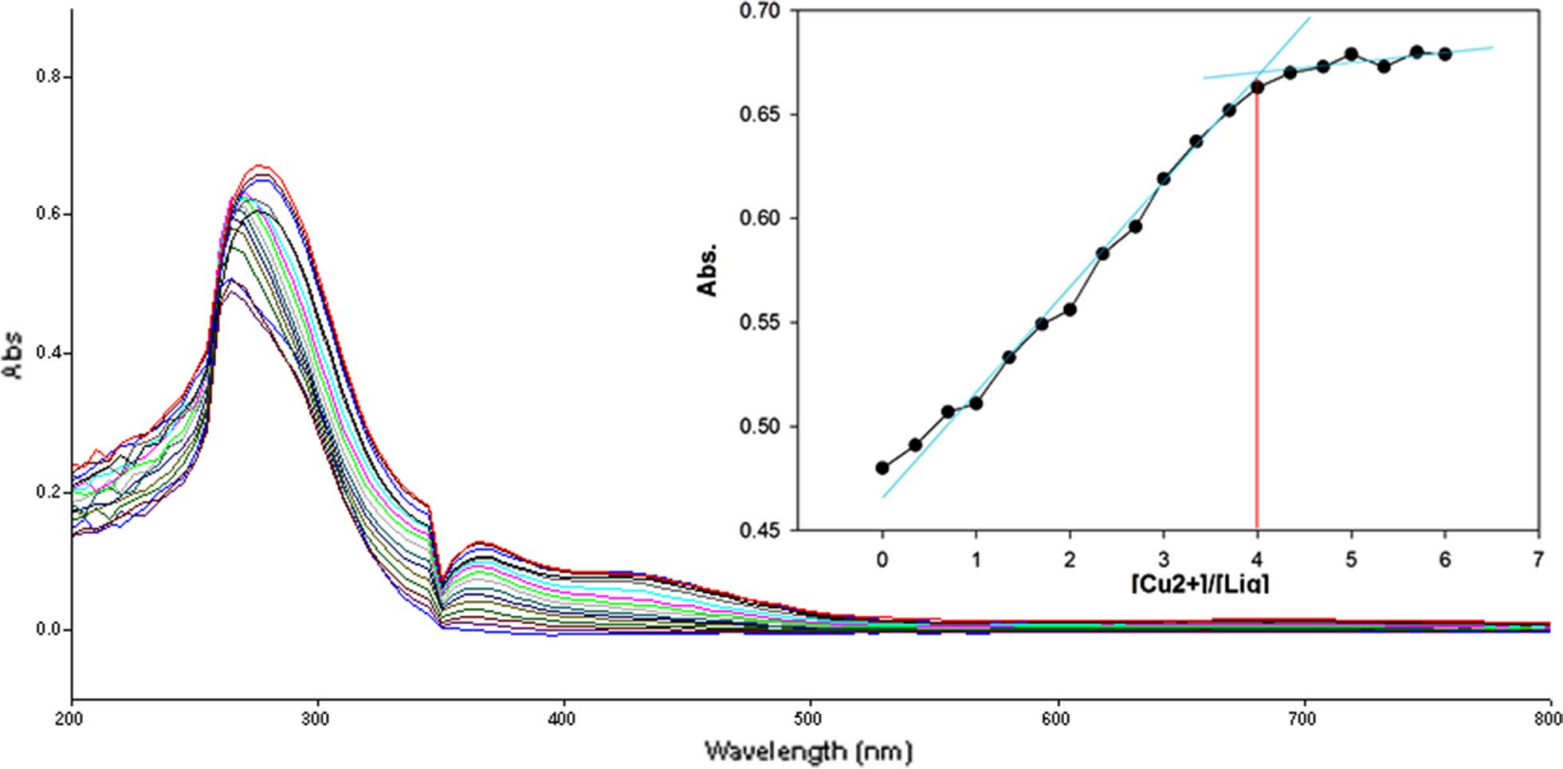
Titration of isomer **1A** vs **Cu** ^**2+**^ (*Inset* Binding behavior + stoichiometry)

**Fig. 11:**
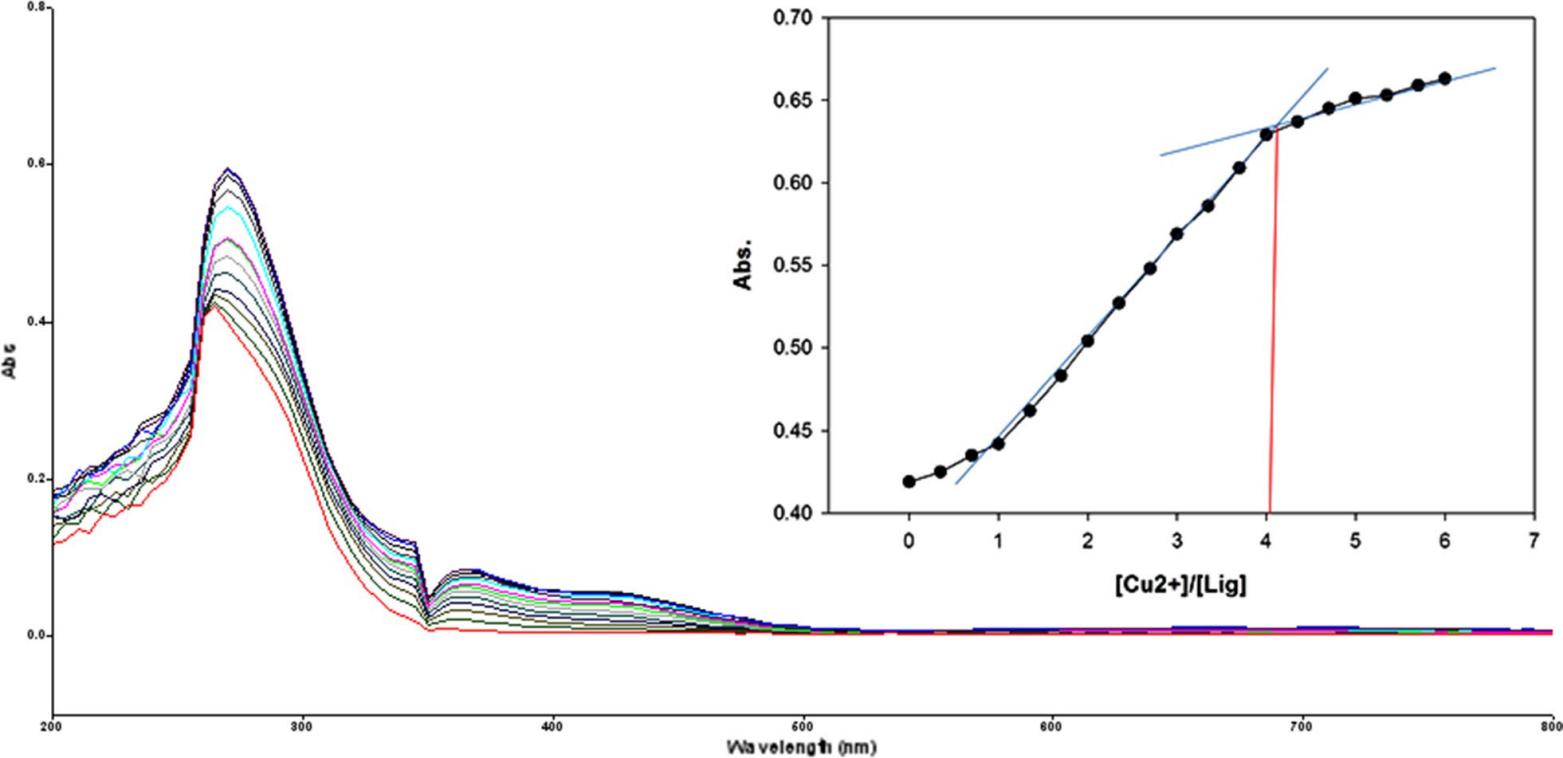
Titration of isomer **1B** vs **Cu** ^**2+**^ (*Inset* Binding behavior + stoichiometry)

**Fig. 12:**
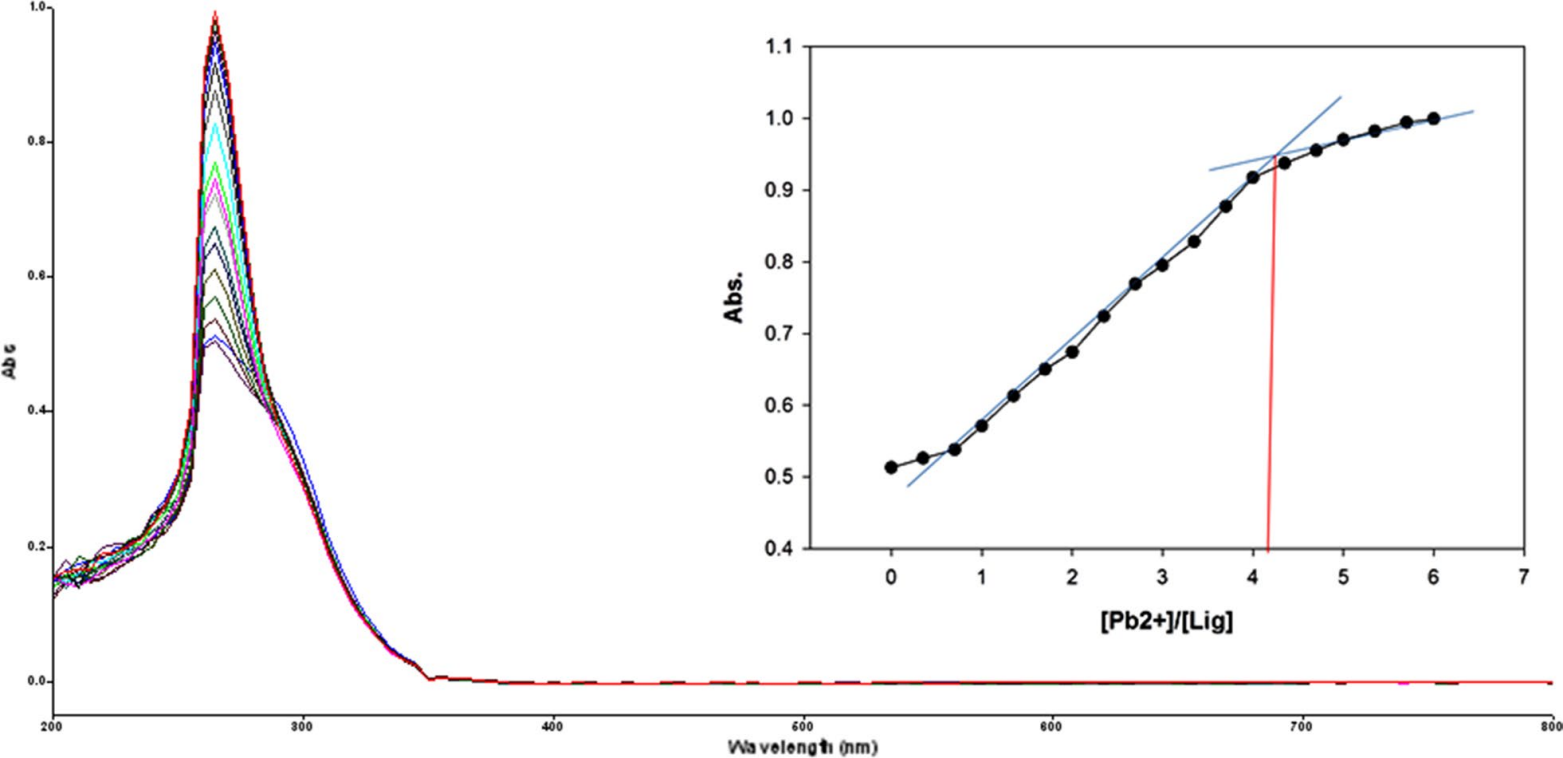
Titration of isomer **1A** vs **Pb** ^**2+**^ (*Inset* Binding behavior + stoichiometry)

**Fig. 13:**
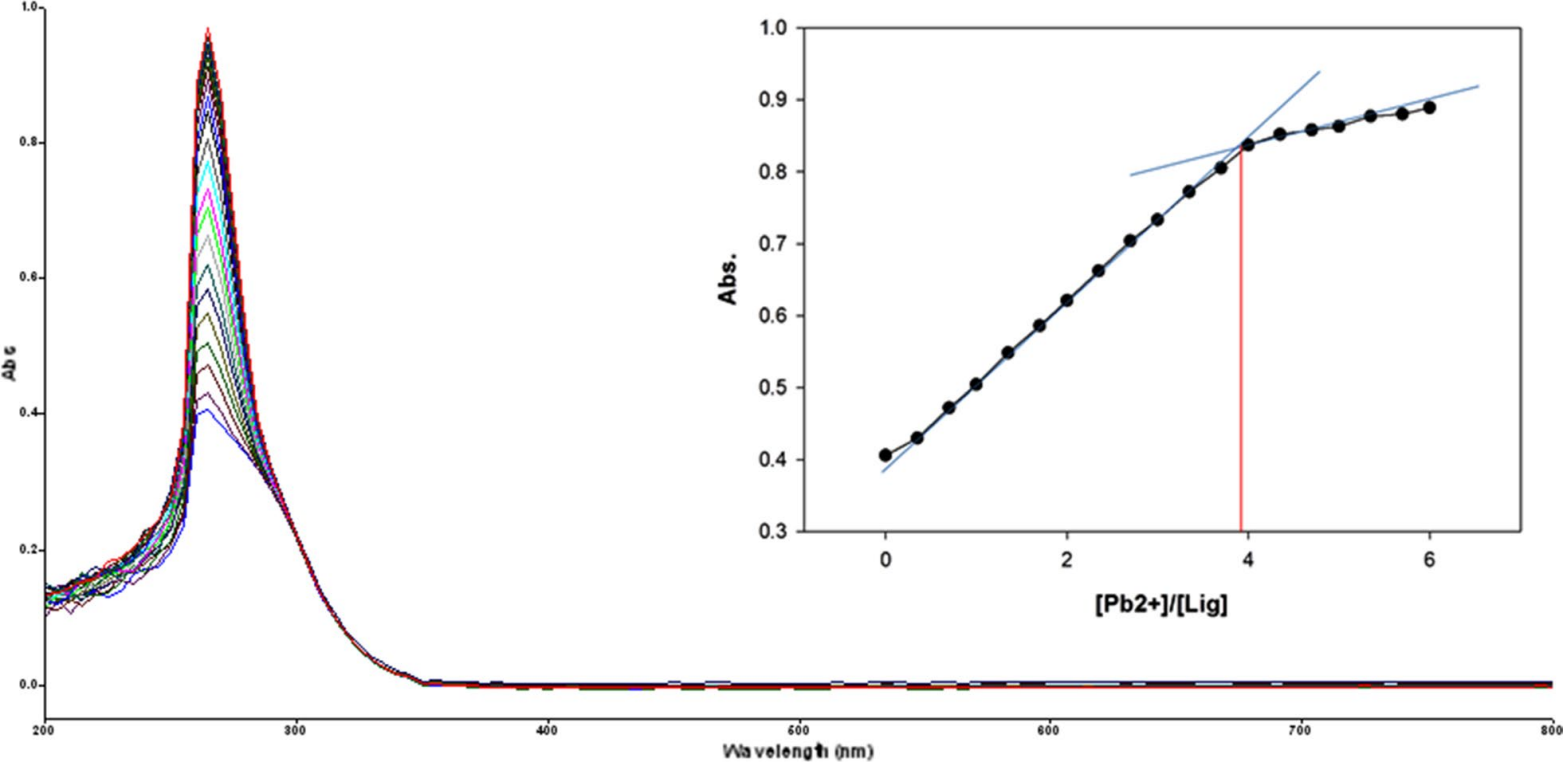
Titration of isomer **1B** vs **Pb** ^**2+**^ (*Inset* Binding behavior + stoichiometry)

**Fig. 14:**
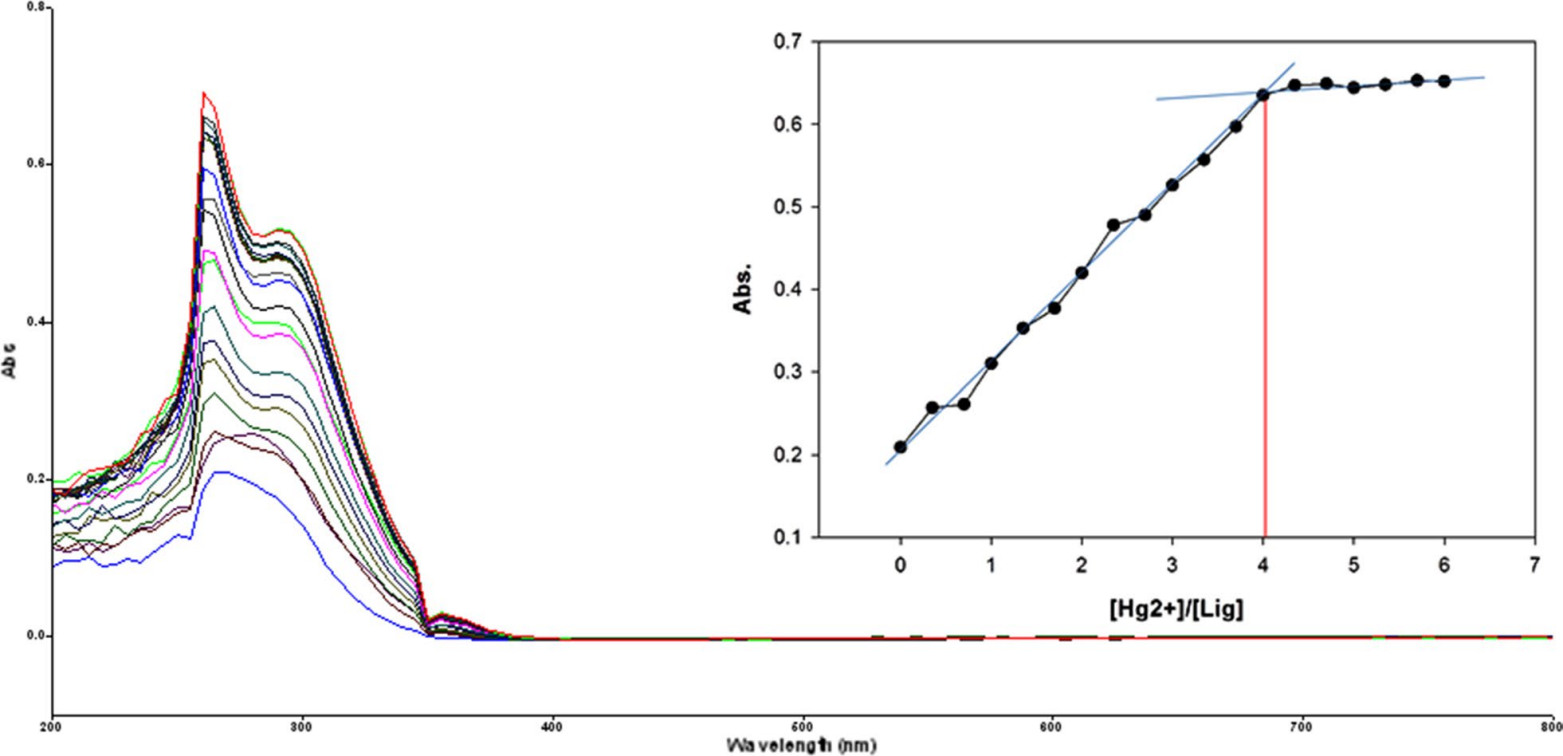
Titration of isomer **1A** vs **Hg** ^**2+**^ (*Inset* Binding behavior + stoichiometry)

**Fig. 15:**
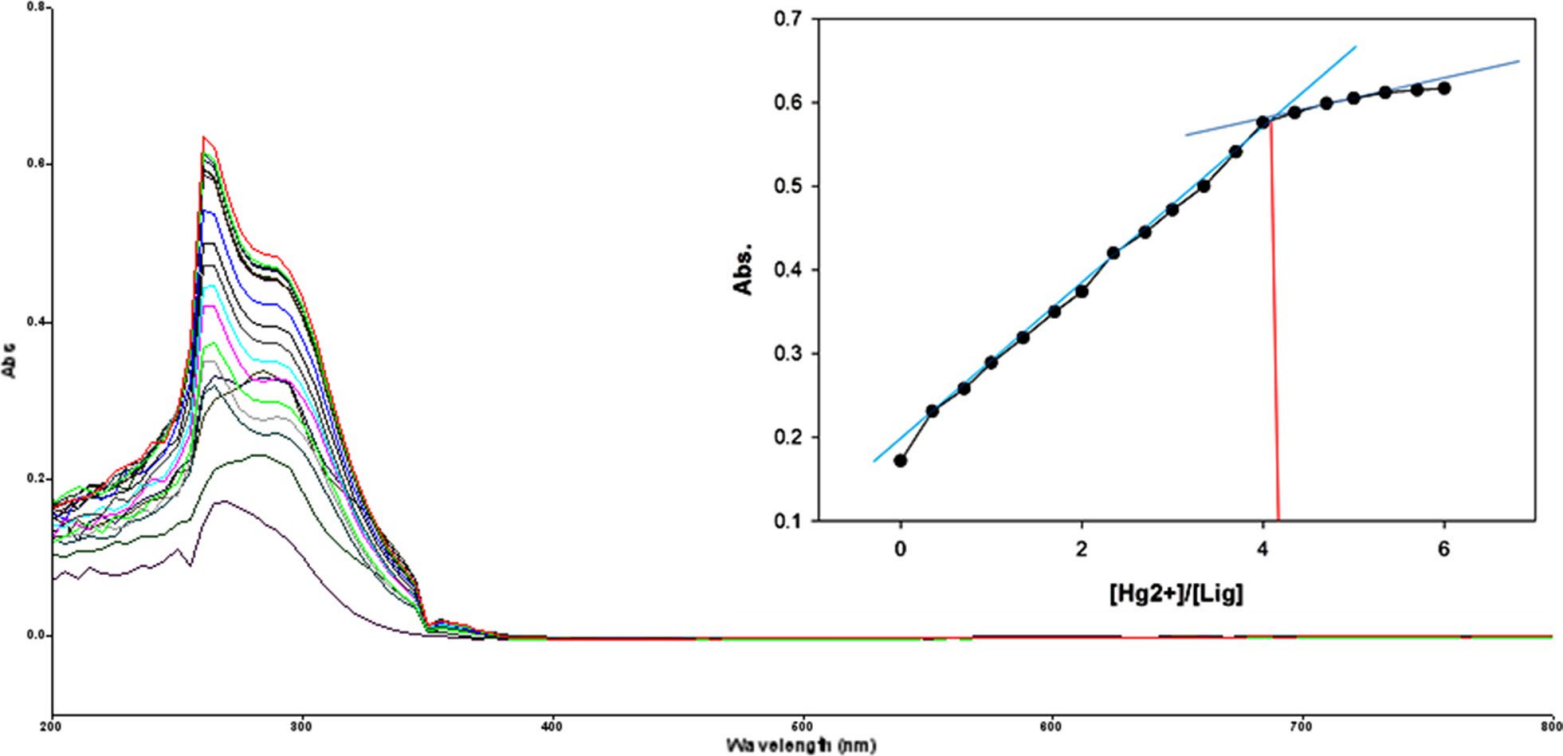
Titration of isomer **1B** vs **Hg** ^**2+**^ (*Inset* Binding behavior + stoichiometry)

**Fig. 16:**
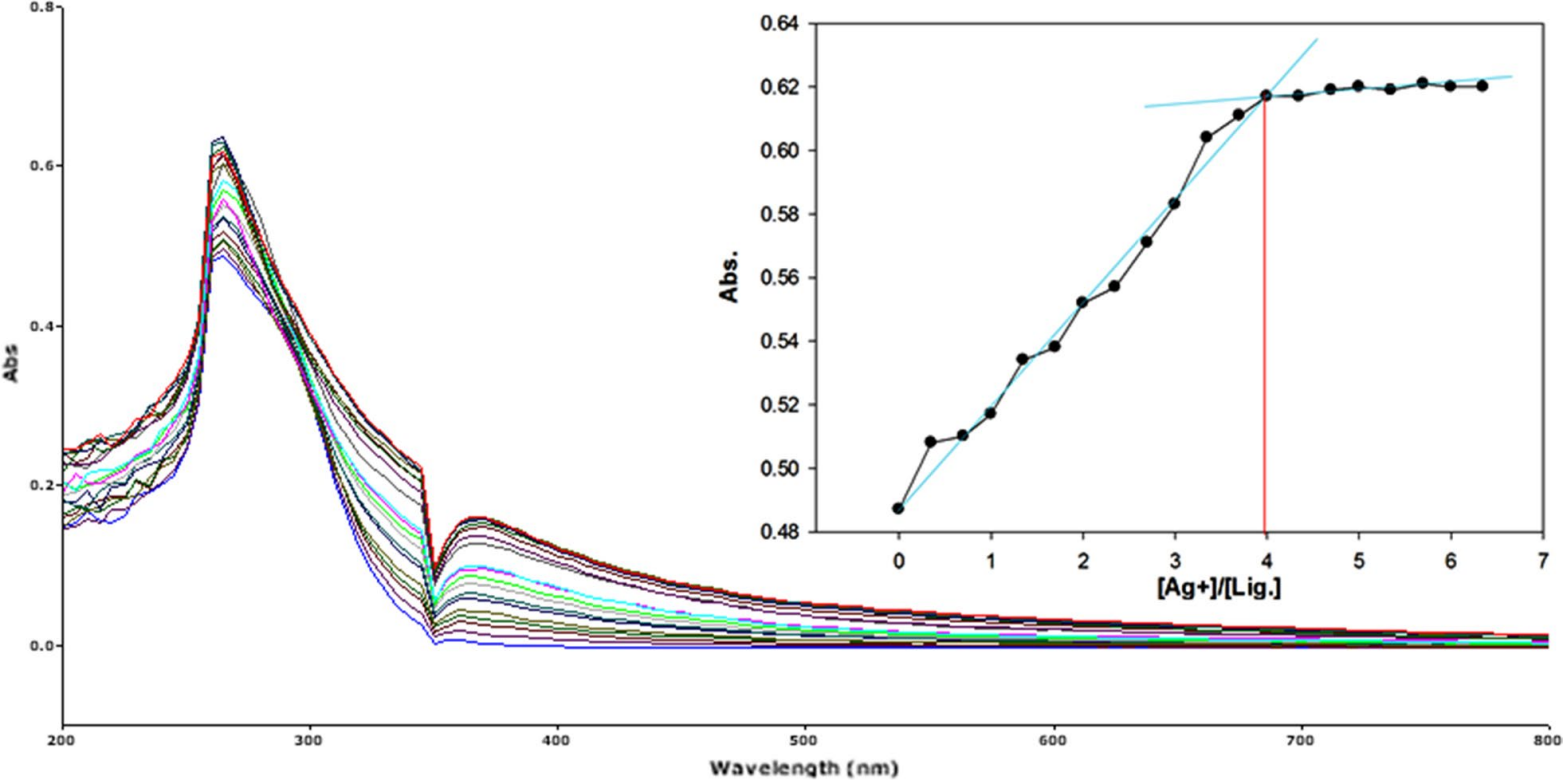
Titration of isomer **1A** vs **Ag** ^**+**^ (*Inset* Binding behavior + stoichiometry)

**Fig. 17:**
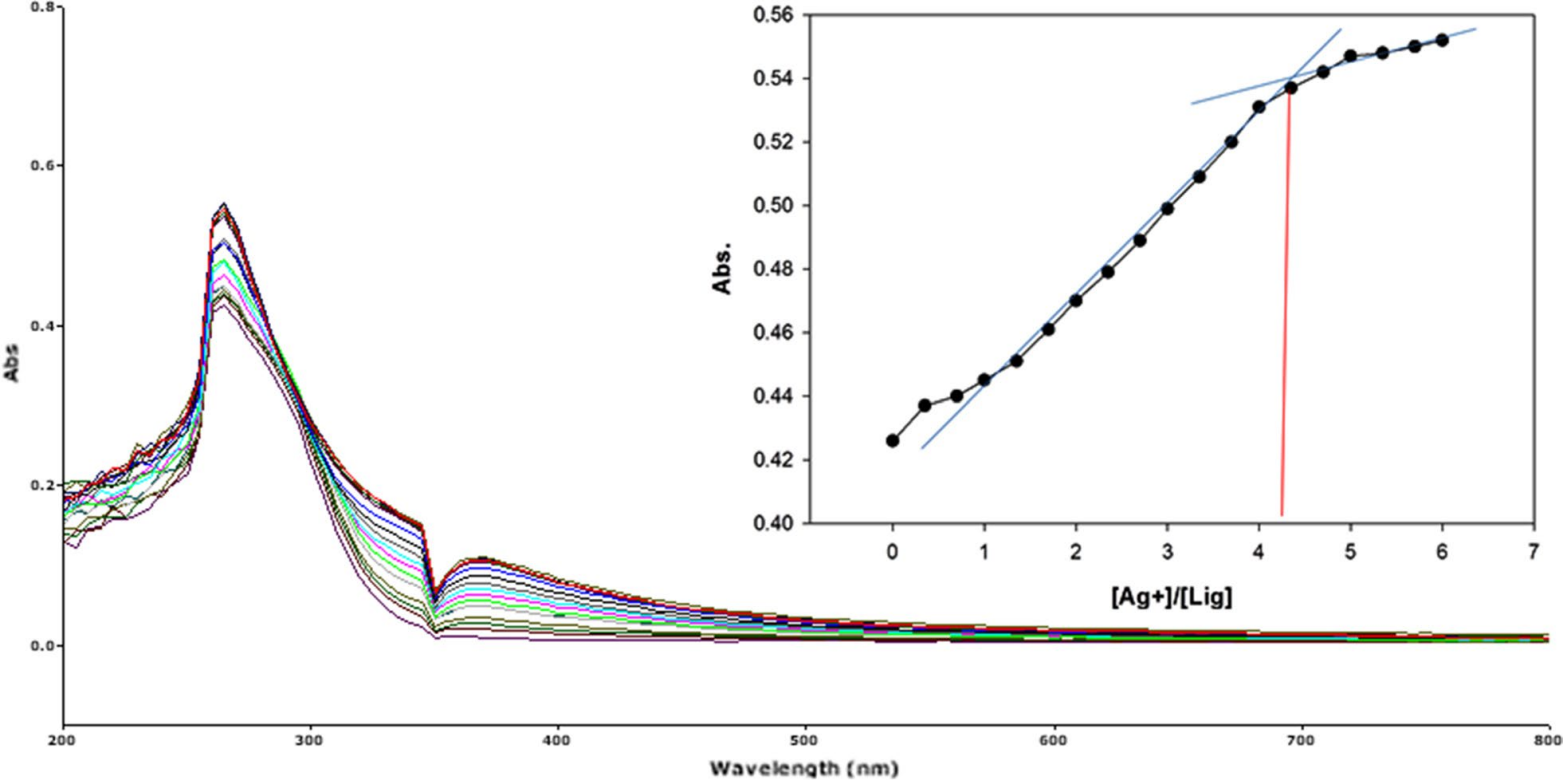
Titration of isomer **1B** vs **Ag** ^**+**^ (*Inset* Binding behavior + stoichiometry)

To explore the mechanism of complexation between isomers 1A and 1B and the chosen metal cations, continuous variation titration investigations were carried out. In these tests, the concentration of cations was increased incrementally, whereas the concentration of isomer 1A and 1B were kept constant. In the light of these titration investigations, the stoichiometry of complexation between isomer 1A/1B with metal cations were ascertained by a molar-ratio strategy [45], and the binding constant (K_d_) computed by nonlinear regression formula [28]. The dissociation constant (K_d_) values and stoichiometry of the complexation are shown in Table 4. The graphical counts of the stoichiometry are also shown (Inset: Figs. 6, 7, 8, 9, 10, 11, 12, 13, 14, 15, 16, 17).

**Table 4:** Correlation coefficient, detection limit, stoichiometry of complexation and binding constants of both Isomers with metal ions

Lig-metal ion	Correlation coefficient	Detection limit	Complexation stoichiometry	Dissociation constant	
				Kd1	Kd2
Isomer1A-Fe^3+^	0.982	1.30 × 10^−1^ M	1:4	5.45 × 10^−17^ M	6.760 M
Isomer1B-Fe^3+^	0.967	2.40 × 10^−1^ M	1:4	3.81 × 10^−17^ M	4.539 M
Isomer1A-Fe^2+^	0.998	1.50 × 10^−1^ M	1:4	1.42 × 10^−18^ M	6.835 M
Isomer1B-Fe^2+^	0.936	3.88 × 10^−1^ M	1:4	1.15 × 10^−17^ M	7.380 M
Isomer1A-Cu^2+^	0.998	1.90 × 10^−1^ M	1:4	6.04 × 10^−18^ M	6.149 M
Isomer1B-Cu^2+^	0.967	3.16 × 10^−1^ M	1:4	5.92 × 10^−17^ M	9.852 M
Isomer1A-Pb^2+^	0.982	1.14 × 10^−1^ M	1:4	2.84 × 10^−17^ M	1.269 M
Isomer1B-Pb^2+^	0.977	1.83 × 10^−1^ M	1:4	5.69 × 10^−17^ M	5.310 M
Isomer1A-Hg^2+^	0.967	9.16 × 10^−2^ M	1:4	9.57 × 10^−17^ M	5.201 M
Isomer1B-Hg^2+^	0.98	2.10 × 10^−1^ M	1:4	5.56 × 10^−18^ M	7.916 M
Isomer1A-Ag^+^	0.997	2.02 × 10^−1^ M	1:4	6.87 × 10^−18^ M	4.557 M
Isomer1B-Ag^+^	0.989	7.23 × 10^−1^ M	1:4	1.64 × 10^−17^ M	1.717 M

## Conclusions

Bis-thiourea isomers featuring amino acids (α and β-alanine) have been successfully characterized using spectroscopic methods, namely; IR, ^1^H NMR, ^13^C NMR, ESI–MS, and elemental analysis (CHNS/O). Moreover, isomer 1B was further confirmed by X-ray crystallography, which revealed that the β-alanine side chain is arranged in a cis–trans configuration. The spectroscopic results also revealed that both isomers exhibit a plane of symmetry. The titration experiments confirmed the interaction of six metal ions; one ‘hard’ acid Fe^3+^, and five ‘soft’ acids Fe^2+^, Cu^2+^, Pb^2+^, Hg^2+^ and Ag^+^. All the remaining metal ions examined (Na^+^, Ca^2+^, Mg^2+^, Co^2+^, Ni^2+^, Mn^2+^, Cd^2+^, Sn^2+^, Zn^2+^, and Al^3+^) showed no appreciable interactions. In addition, no interaction was observed for the tetrabutylammonium ions Cl^−^ and H_2_PO_4_^−^. The stoichiometry of the complex (host–guest)formed for both isomers was found to be 1:4. Binding constant K_d1_ values for both isomers were found to be very low due to complexation at carboxylate functionality of α and β-alanine. Binding constant (K_d2_) values were appreciably high as compared to K_d1_ values because of the complexation at the thiourea functionality of isomers 1A and 1B. On comparing the binding constant (Kd_2_) for both isomers, values for isomer 1A were in the range 4.5–6.8 except for (Pb^2+^) which was 1.2 and for isomer 1B binding constant values were found in the range 4.5–9.8 except for (Ag^+^) which was 1.7. The dissociation constant values for both isomers with all metal ions were in relatively close proximity to each other. The next study will be focused on the role of different side chain amino acids/secondary amines towards binding behavior against various metals and based on the data obtained in the present study, the chemical sensor will be fabricated by using newly synthesized compounds for the detection of metal ions.
